# Influencing factors and network analysis of latent profiles of care demands among disabled older adults in integrated medical and nursing care institutions in Shanghai, China: a cross-sectional study

**DOI:** 10.3389/fpubh.2026.1938348

**Published:** 2026-10-01

**Authors:** Xu Ao, Sun Haijing, Sun Hongmei, Wei Lihua, Wang Yanmei

**Affiliations:** 1 School of Nursing, Ningxia Medical University, Yinchuan, China; 2 Department of Nursing, Pudong Gongli Hospital, Shanghai University of Medicine and Health Sciences, Shanghai, China

**Keywords:** care demands, disabled older adults, influencing factors, latent profile analysis, network analysis, nursing institutions

## Abstract

**Objective:**

To identify the latent profiles and network structure of care demands among disabled older adults in integrated medical and nursing care institutions, and to provide empirical evidence for service provision.

**Methods:**

Convenience sampling was used to select 443 disabled older adults from 16 integrated medical and nursing care institutions in Pudong New Area, Shanghai. Face-to-face surveys were conducted using a general information questionnaire and a service demands questionnaire. Latent profile analysis was employed to identify care demand profiles and their influencing factors, while network analysis was applied to construct the demand network and compare network differences across profiles.

**Results:**

Latent profile analysis identified three care demand profiles: the “Low-demand expression group” (76.4%), the “Multidimensional high-demand group” (18.5%), and the “Psychological-prominent demand group” (5.1%). Age, gender, pre-retirement occupation, and frequency of children’s visits were independent influencing factors for profile membership (*P*<0.05). The overall network analysis revealed that chronic disease management health education and safety prevention health education served as bridge demands. In both the overall and subgroup network analyses, excretion care and rehabilitation assessment were core demands. Subgroup comparison showed that the strongest edge in the “Low-demand expression group” crossed dimensions, whereas the strongest edge in the “Multidimensional high-demand group” was concentrated within dimensions.

**Conclusion:**

The care demands of disabled older adults in integrated medical and nursing care institutions are typologically differentiated and shaped by multiple factors. The association structures among care demands differ fundamentally across demand types. Therefore, institutions should consider both the demand classification and network structures when allocating care resources, rather than determining service priorities based solely on the average scores of individual demand items.

## 1 Introduction

Global population aging is occurring at an unprecedented rate. According to the World Health Organization (WHO), the number of people aged 60 years and older worldwide is projected to reach 2.1 billion by 2050, among whom those aged 80 years and older will increase from 141 million in 2020 to 426 million by 2050 (1). Disability represents one of the core manifestations of health decline in older adults. The WHO estimates that approximately 15.6% of the global population (around 1 billion people) live with varying degrees of disability, and this proportion rises markedly with advancing age. United Nations data further indicate that over 46% of the global population aged 60 years and older experience some form of disability, with more than 250 million individuals experiencing moderate to severe disability (2). A systematic review and meta-analysis published in 2025, pooling data from 133,827 older adults worldwide, reported a pooled prevalence of 26.07% for disability in basic activities of daily living (ADL) and 45.15% for disability in instrumental activities of daily living (IADL) (3). Across Europe, North America, East Asia, and other regions with advanced population aging, the care demands of disabled older adults have become a shared challenge confronting health systems and social welfare frameworks. Disabled older adults refer to those who, due to natural aging or chronic diseases, are partially or completely unable to care for themselves and require assistance from others. Their functional impairments are primarily characterized by limited mobility, cognitive dysfunction, diminished self-care capacity, and restricted social participation (4). Their care demands are notably multidimensional and complex, encompassing multiple interrelated domains such as medical care, daily living assistance, rehabilitation, and psychological support (5). In terms of medical care, medication management, disease monitoring, scheduled clinical visits, and complication prevention have become fundamental components of routine care (6). Most disabled older adults suffer from multiple chronic conditions, carrying a heavy disease burden and facing an elevated risk of functional decline (7); some individuals with severely limited mobility require long-term bed rest, accompanied by challenges in continence management, respiratory support, and repositioning, which demands a relatively high level of professional knowledge and practical skills from caregivers. At the psychological level, disabled older adults frequently experience emotional issues such as loneliness, anxiety, or depression (8, 9), demonstrating considerable demands for emotional companionship, social interaction, and psychological support.

In China, along with persistently low fertility levels, the family structure has exhibited trends toward nuclearization, miniaturization, and an increase in empty-nest households, leading to a gradual weakening of familial care functions (10). Consequently, a growing number of disabled older adults are compelled to rely on professional long-term care institutions for round-the-clock, comprehensive care services. Some studies indicate that over 80% of older adults residing in institutions are living with a disability (11, 12). The service capacity of long-term care institutions is directly associated with the health outcomes and quality of life of this population. In response to the dual medical and long-term care demands of disabled older adults, integrated medical and nursing care institutions, possessing both qualified medical and health resources and older adult care service capabilities, have emerged (13). By integrating medical treatment, nursing care, rehabilitation, daily care, and psychological support, these institutions deliver continuous and comprehensive care to disabled older adults. Nevertheless, the implementation of integrated medical and nursing care services continues to confront mismatches between service supply and care demands in practice (14). Due to regional disparities in economic development, the distribution of health resources, and the intensity of policy implementation, institutions differ considerably in medical support capacity, professional staffing, and service standardization (15). While some long-term care institutions are able to provide relatively comprehensive medical and nursing care, a substantial number remain confined to basic daily care, lacking the necessary medical qualifications and technical support (16). Evidence from China further suggests that institutional ownership and operation may be associated with differences in service performance. A multicity study reported differences in the quality of integrated medical and nursing services between public and private nursing institutions (15). This structural heterogeneity on the supply side, together with heterogeneous resident needs, highlights the need to identify care-demand patterns and the structural relationships among individual demands within institutional settings.

Current research on the care demands of older adults in long-term care institutions has predominantly employed descriptive statistical analyses or regression-based approaches, focusing on the mean levels of individual demand items and their associated factors (17–20). Methodologically, variable-centered approaches estimate average associations among variables and generally apply a common population-level structure. They are well suited to questions about average effects, but may obscure meaningful subgroups when individuals differ in the configurations of multiple needs. Person-centered approaches developed as a complementary framework that shifts the analytical focus from isolated variables to patterns within individuals (21). In research on older adults with complex care needs, latent-class approaches have shown that multidimensional health and care-related characteristics can form clinically meaningful profiles that are not adequately represented by single-variable averages (22). This methodological shift is particularly relevant for disabled older adults, whose daily living, medical, rehabilitation, and psychological needs may cluster in different combinations. In addition, individual care demands may be interconnected rather than independent. Latent profile analysis can identify unobserved heterogeneous subgroups based on multidimensional indicators, whereas network analysis can quantify conditional relationships among demand items and identify core and bridge demands (23). Integrating these two approaches therefore enables both the stratification of care demands and the examination of their internal association patterns within different profiles; however, this combined framework has not yet been applied to disabled older adults in integrated medical and nursing care institutions.

Pudong New Area, the most populous administrative district in Shanghai, had 1.1368 million residents aged 60 years or older by the end of 2025, the highest number among all districts in the municipality (24). The district is also characterized by an active policy environment for integrated medical and nursing care and a diverse range of institutional ownership and operation models. Against this context, this study focused on disabled older adults residing in integrated medical and nursing care institutions in Pudong New Area. First, latent profile analysis was used to identify latent profiles of multidimensional care demands and characterize population heterogeneity. Second, factors associated with profile membership were examined to further clarify differences among demand subgroups. Third, network analysis was conducted to identify core and bridge demands and to compare the internal demand structures across latent profiles. This study aimed to provide empirical evidence for more precise identification of care demands and more targeted allocation of care resources in integrated medical and nursing care institutions.

## 2 Subjects and methods

### 2.1 Research subjects

Using convenience sampling, disabled older adults were selected from 16 integrated medical and nursing care institutions in Pudong New Area, Shanghai (7 publicly built and privately operated, 7 privately built and privately operated, and 2 publicly built and publicly operated) from January 2026 to May 2026. Inclusion criteria: ① Age ≥60 years; ②Able to communicate normally with intact cognitive ability; ③ Residing in the same integrated medical and nursing care institution for ≥6 months; ④ According to the “Specification for Ability Assessment of Older Adults” (25), total score <90; ⑤ Provided informed consent and voluntarily participated (for those unable to communicate, consent was obtained from the primary caregiver). Exclusion criteria: ① Primary caregiver refused to participate; ② Unable to cooperate with the survey due to illness, cognitive impairment, or other reasons.

In logistic regression, the sample size is typically set such that each predictive variable corresponds to a certain number of events (events per variable, EPV), with at least 10 events per variable (EPV ≥ 10) (26) (this study had 19 predictive variables). However, according to existing research, latent profile analysis (LPA) generally recommends a minimum sample size of 300 (27). Additionally, the sample size for network analysis (28) = *N* + *N* × (*N*-1) ÷ 2, where N is the number of nodes (*N* = 23 in this study). Considering a 1% invalid questionnaire rate (29) (face-to-face interviews were used in this study), the final calculated minimum sample size was at least 303. A total of 451 questionnaires were distributed, and after excluding those with obvious logic errors, 443 valid questionnaires were ultimately collected, with an effective response rate of 98.23%.

### 2.2 Survey instruments

#### 2.2.1 General information questionnaire

Self-designed based on literature review, comprising 19 predictive variables: type of older adult care institution, grade of older adult care institution (30), care level of the older adult (31), age, gender, educational level, religious belief, marital status, pre-retirement occupation, source of income, personal monthly income, number of chronic diseases, number of medications taken, length of stay in the institution, reason for admission, number of living children, gender of living children, frequency of children’s visits, and type of basic medical insurance.

#### 2.2.2 Service supply-demands matching questionnaire for disabled older adults in integrated medical and nursing care institutions (demands subscale)

This questionnaire, developed by Xu Lidan et al. (32), is designed to assess the care demands of disabled older adults in integrated medical and nursing care institutions. It comprises four dimensions: daily living care, medical care, rehabilitation care, and psychological solace, with a total of 23 items. A Likert 5-point scale is used, ranging from “not demandsed” to “very demandsed,” scored from 1 to 5, with higher scores indicating greater demands. The questionnaire demonstrated good content validity (*I-CVI* = 0.929, *S-CVI* = 0.957) and reliability (Cronbach’s α coefficient for the demands subscale = 0.910). In this study, the Cronbach’s α coefficient for the demands subscale was 0.806, indicating good reliability.

### 2.3 Data collection methods and quality control

A questionnaire collection team was established (2 graduate students and 2 undergraduate students). Before the formal survey, a pilot survey was conducted with 30 disabled older adults to test the applicability of the demands questionnaire and the feasibility of the survey process (33). The revised questionnaire was converted into an electronic questionnaire using the Wenjuanxing platform. Contact was made with the administrators of each integrated medical and nursing care institution to explain the purpose, significance, and completion methods of the survey. Prior to the formal survey, the researchers organized unified training covering research objectives, inclusion and exclusion criteria, interview techniques, informed consent procedures, and electronic questionnaire completion standards. After participants signed paper informed consent forms, survey personnel conducted face-to-face interviews and completed the questionnaires. Surveys were conducted in private settings to minimize distractions, and suggestive language was avoided during the process.

### 2.4 Statistical methods

Data for this study were derived from electronic questionnaires, with all items set as mandatory. The online system directly exported data in Excel format, and there were no missing values. After export, outliers were corrected or deleted based on original records and logic checks. Statistical analyses were performed using R 4.4.3 (RStudio 2024.12.0) and Mplus 9.0.

#### 2.4.1 Description of general information

Count data are presented as frequencies and constituent ratios. Measurement data were tested for normality using the Shapiro–Wilk test. Normally distributed quantitative data are presented as mean ± standard deviation (*X̄*±*SD*), while non-normally distributed variables are presented as median (interquartile range) [*M (P_25_, P_75_)*].

#### 2.4.2 Latent profile analysis

The mean scores of each demands dimension were used as observed indicators, and latent profile analysis was conducted using Mplus 9.0. The number of profiles was increased progressively from one. The optimal model was determined by comprehensively evaluating the Akaike Information Criterion (*AIC*), Bayesian Information Criterion (*BIC*), adjusted BIC (*aBIC*), entropy, Lo–Mendell–Rubin likelihood ratio test (*LMRT*), and bootstrap likelihood ratio test (*BLRT*). Smaller AIC, BIC, and aBIC values and entropy values closer to 1 indicated better model fit. LMRT and BLRT with *p* < 0.05 indicated that a k-profile model was superior to a *k*-1 profile model. *T*-tests, non-parametric tests, chi-square tests, or one-way ANOVA were used to compare differences in general information across profiles, with *P*<0.10 considered statistically significant for screening predictive variables. Using profile as the dependent variable, the predictive variables screened by univariate analysis were included in multinomial logistic regression to analyze the independent associations between profiles and predictive variables, with *P*<0.05 considered statistically significant.

#### 2.4.3 Demands network analysis

The qgraph package was used to construct the demands network, with nodes representing the 23 care demands items, grouped into four clusters: daily living care, medical care, rehabilitation care, and psychological solace. Edge weights were calculated based on Spearman correlations, and the EBICglasso function was used to select edges, with layout determined by the Fruchterman-Reingold algorithm. The mgm package was used to calculate the coefficient of determination (*R^2^*) for each node, assessing the extent to which a node is predicted by other nodes in the network. The circular fill around each node represents the proportion of variance explained by other nodes in the network. Blue lines between nodes indicate positive correlations, red lines indicate negative correlations, and thicker edges indicate stronger correlations. The qgraph and networktools packages were used to calculate strength centrality, expected influence, and bridge expected influence (*BEI*). Strength centrality is the sum of the absolute values of a node’s edge weights; expected impact sums edge weights while retaining their signs, reflecting the direction and magnitude of net impact (23). The bootnet package was used for stability testing, with non-parametric bootstrapping (1,000 resamples) to estimate 95% confidence intervals for edge weights and case-dropping bootstrapping to calculate the correlation stability coefficient (CS coefficient). CS coefficients should be >0.25, with >0.50 considered preferable (34). For network analysis, *p* < 0.05 was considered statistically significant.

## 3 Results

### 3.1 Care demands of disabled older adults in integrated medical and nursing care institutions

Table 1 summarizes the scores of care demands across the four dimensions and 23 individual items.

**Table 1:** Scores of care demands of disabled older adult in medical-nursing combined pension institutions (*n* = 443).

Demands type	Demands level (score)
Daily living care dimension	2.00 (1.43, 2.93)
LC1 Provide daily living services for you	3.00 (2.00, 4.00)
LC2 Provide cleaning care services for you	3.00 (2.00, 4.00)
LC3 Assist you with eating and drinking, or feeding via nasal feeding tube, etc.	1.00 (1.00, 2.00)
LC4 Assist you with toileting, using and cleaning bedpans, or timely changing diapers	1.00 (1.00, 3.00)
LC5 Create a quiet sleeping environment for you and respond promptly when you are insomnia or demands help at night	2.00 (1.00, 3.00)
LC6 Guide and assist you in correctly using walking aids, wheelchairs, and other mobility aids	1.00 (1.00, 3.00)
LC7 Provide basic nursing services for you	1.00 (1.00, 3.00)
Medical care dimension	3.12 (2.62, 3.38)
MC8 Establish and continuously maintain a detailed and practical personal health record for you	5.00 (4.00, 5.00)
MC9 Provide regular health check-ups and interpretation of examination reports for you	3.00 (2.00, 4.00)
MC10 Provide comprehensive disease management services (chronic disease monitoring and common disease diagnosis and treatment) for you	3.00 (3.00, 4.00)
MC11 Timely and effectively manage common geriatric discomforts such as low back pain, insomnia, and constipation	3.00 (2.00, 4.00)
MC12 Ensure absolute safety and correctness of medication (dedicated management, timely reminders, monitoring of side effects)	3.00 (2.00, 4.00)
MC13 Provide convenient referral and emergency medical rescue services for you	5.00 (4.00, 5.00)
MC14 Provide health education on safety prevention for older adults	3.00 (2.00, 3.00)
MC15 Assist you in maintaining good contact and communication with family members	1.00 (1.00, 1.00)
Rehabilitation care dimension	2.00 (1.40, 3.00)
RC16 Conduct rehabilitation assessment and regular evaluation for motor, social, and psychological aspects	2.00 (1.00, 3.00)
RC17 Provide one-on-one personalized rehabilitation knowledge education and guidance services for you	2.00 (1.00, 3.00)
RC18 Provide rehabilitation training in limb movement, muscle strength, speech, gait, hearing, etc.	2.00 (1.00, 3.00)
RC19 Provide traditional Chinese medicine conditioning services for you (moxibustion, massage, etc.)	2.00 (1.00, 3.00)
RC20 Provide health education on chronic disease management for older adults	3.00 (1.00, 3.00)
Psychological comfort dimension	1.33 (1.00, 2.00)
PC21 Arrange professional psychological counseling or intervention when feeling depressed, anxious, or distressed	1.00 (1.00, 2.00)
PC22 Conduct regular psychological status screening through questionnaires to identify risks early and provide preventive guidance	1.00 (1.00, 2.00)
PC23 Staff frequently take the initiative to chat with you, providing daily companionship and care	1.00 (1.00, 2.00)

LC = daily living care; MC = medical care; RC = rehabilitation care; PC = psychological comfort. Data are presented as median (P25, P75) due to non-normal distribution.

### 3.2 Latent profile analysis of care demands of disabled older adults

Using the mean scores of each questionnaire dimension as observed indicators, latent profile analysis was conducted, starting from the initial model and completing a total of five latent profile models, as shown in Table 2. As the number of profiles increased, AIC, BIC, and aBIC gradually decreased. The LMR test and BLRT for models with 2 to 5 profiles all reached significant levels (*P*<0.05), and entropy values were all greater than 0.8, indicating progressively improved model fit. However, model selection cannot rely solely on statistical significance; it also requires a comprehensive evaluation of parsimony, theoretical interpretability, and downstream analytic feasibility. Specifically, although the 4-profile model showed a significant LMRT (*p* = 0.0175) and continued improvement in fit indices, its smallest class accounted for only 3.4% of the total sample (approximately 15 individuals). More importantly, close examination of the mean profiles revealed that the 4-class solution did not produce a qualitatively new subtype, but merely subdivided the “Multidimensional high-demand group” from the 3-class solution along a quantitative gradient, offering limited clinical added value. For the 5-profile model, Mplus explicitly issued a warning that “THE BEST LOGLIKELIHOOD VALUE WAS NOT REPLICATED”, indicating an untrustworthy solution potentially affected by local maxima. Therefore, based on comprehensive statistical indicators, clinical interpretability, and the sample size requirements for subsequent subgroup network analyses, the 3-profile latent profile model was finally determined as the optimal model. Profile characteristics were plotted based on the results, as shown in Figure 1. Profiles were named according to the characteristics of the mean scores across dimensions for each profile. Profile 1 (76.4%) had the first three demands dimensions significantly lower than Profile 2, and psychological solace demands also significantly lower than Profiles 2 and 3, with the lowest overall demands, thus named “Low-demand expression group”; Profile 2 (18.5%) had the highest scores in the first three dimensions among the three groups, with psychological solace demands in the middle, thus named “Multidimensional high-demand group”; Profile 3 (5.1%) was characterized by prominently elevated and independent psychological solace demands, thus named “Psychological-prominent demand group.”

**Table 2:** Fit indices of latent profile models for care demands of disabled older adults in integrated medical and nursing care institutions.

Model	AIC	BIC	aBIC	Entropy	LMR (P)	BLRT (P)	Class probability (%)
1	4437.100	4469.848	4444.460	1.000			
2	4219.652	4272.869	4231.612	0.954	0.0002	<0.001	90.4/9.6
3	4111.796	4185.481	4128.357	0.916	0.0063	<0.001	76.4/18.5/5.1
4	3992.748	4086.900	4013.909	0.931	0.0175	<0.001	7.6/22.6/66.5/3.4
5	3934.138	4048.758	3959.899	0.964	0.0021	<0.001	61.8/19.7/6.3/3.4/8.7

**Figure 1:**
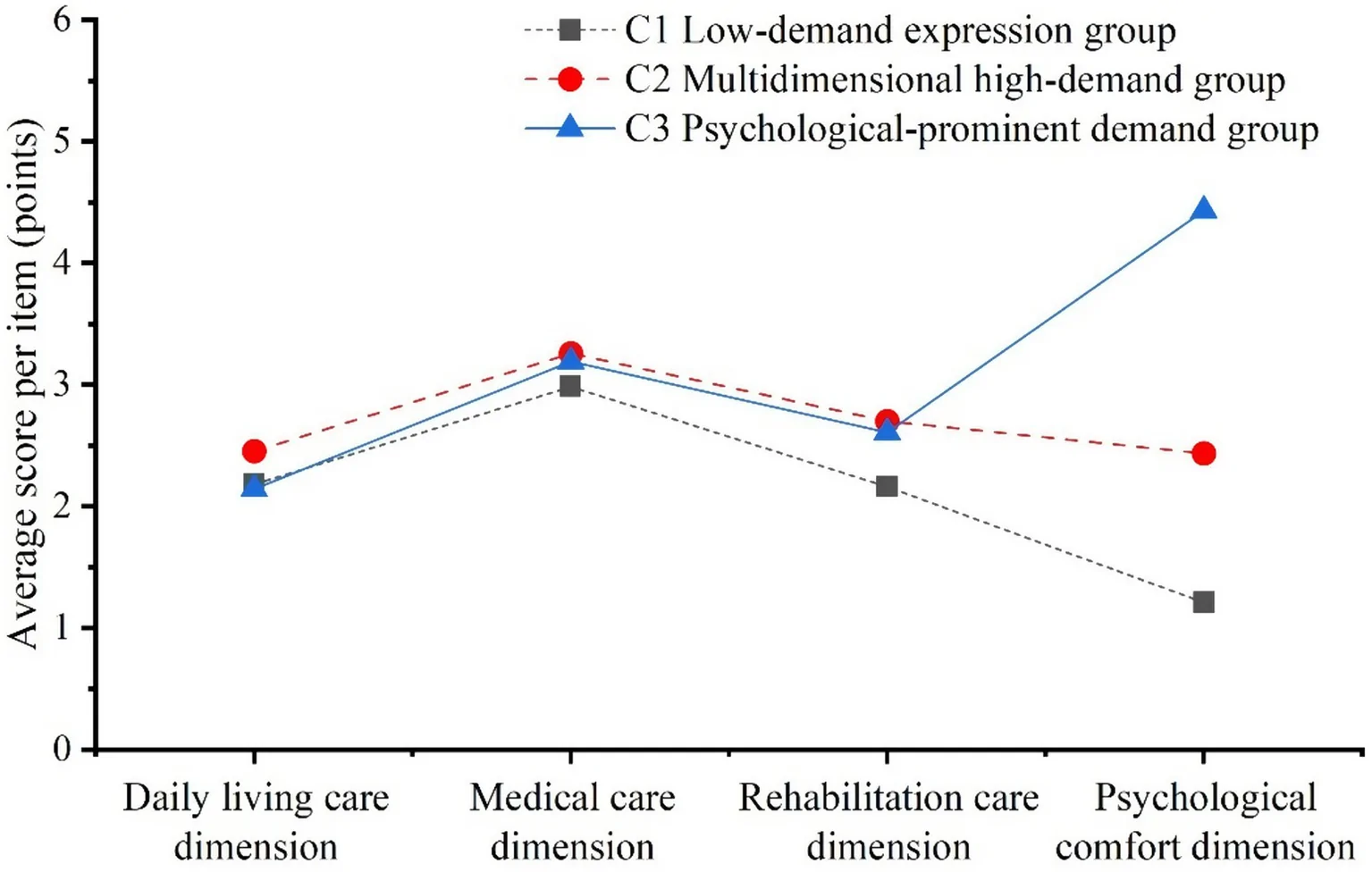
Latent profile characteristic distribution of care demands of disabled older adults in integrated medical and nursing care institutions.

### 3.3 Univariate analysis of factors influencing care demands profiles of disabled older adults

Univariate analysis showed no statistically significant differences across latent profile groups in terms of personal monthly income, grade of older adult care institution, care level, educational level, length of stay in the institution, number of living children, type of older adult care institution, marital status, primary source of income, reason for admission, gender of living children, or type of basic medical insurance. Variables with statistically significant differences are shown in Table 3 (*p* < 0.10).

**Table 3:** Univariate analysis of factors influencing care demands profiles of disabled older adults.

Variable	*n*	C1 (*n* = 334)	C2 (*n* = 86)	C3 (*n* = 23)	Statistic	*p* value
Age (years)	—	87 (82, 90)	88 (83, 92)	81 (78, 89)	H = 10.80	**0.0045**
Personal monthly income (yuan)	—	5,000 (4,000, 6,000)	5,000 (3,250, 7,000)	5,000 (4,750, 6,000)	H = 1.37	0.5039
Grade of older adult care institution					H = 1.04	0.5935
Level 1	4	3 (75.0%)	1 (25.0%)	0 (0.0%)		
Level 2	121	92 (76.0%)	26 (21.5%)	3 (2.5%)		
Level 3	234	181 (77.4%)	36 (15.4%)	17 (7.3%)		
Level 5	84	58 (69.0%)	23 (27.4%)	3 (3.6%)		
Care level of older adult					H = 0.83	0.6610
Mild	80	58 (72.5%)	15 (18.8%)	7 (8.8%)		
Moderate I	149	123 (82.6%)	23 (15.4%)	3 (2.0%)		
Moderate II	78	50 (64.1%)	22 (28.2%)	6 (7.7%)		
Severe I	77	61 (79.2%)	12 (15.6%)	4 (5.2%)		
Severe II	59	42 (71.2%)	14 (23.7%)	3 (5.1%)		
Educational level					H = 3.56	0.1682
Illiterate	75	57 (76.0%)	17 (22.7%)	1 (1.3%)		
Primary school	118	89 (75.4%)	22 (18.6%)	7 (5.9%)		
Middle school	111	87 (78.4%)	20 (18.0%)	4 (3.6%)		
High school/technical secondary	61	51 (83.6%)	7 (11.5%)	3 (4.9%)		
Junior college	36	28 (77.8%)	4 (11.1%)	4 (11.1%)		
Bachelor’s degree	36	18 (50.0%)	14 (38.9%)	4 (11.1%)		
Master’s degree or above	6	4 (66.7%)	2 (33.3%)	0 (0.0%)		
Number of chronic diseases					H = 6.68	**0.0354**
None	30	17 (56.7%)	8 (26.7%)	5 (16.7%)		
1 type	74	60 (81.1%)	11 (14.9%)	3 (4.1%)		
2 types	133	106 (79.7%)	18 (13.5%)	9 (6.8%)		
≥3 types	206	151 (73.3%)	49 (23.8%)	6 (2.9%)		
Number of medications taken					H = 4.92	0.0854
0 types	37	27 (73.0%)	6 (16.2%)	4 (10.8%)		
1–3 types	244	189 (77.5%)	41 (16.8%)	14 (5.7%)		
3–5 types	109	77 (70.6%)	29 (26.6%)	3 (2.8%)		
≥5 types	53	41 (77.4%)	10 (18.9%)	2 (3.8%)		
Length of stay in institution					H = 0.83	0.6609
<1 year	101	74 (73.3%)	19 (18.8%)	8 (7.9%)		
1–2 years	114	85 (74.6%)	25 (21.9%)	4 (3.5%)		
≥2 years	228	175 (76.8%)	42 (18.4%)	11 (4.8%)		
Number of living children					H = 1.34	0.5127
0	15	14 (93.3%)	1 (6.7%)	0 (0.0%)		
1	125	91 (72.8%)	24 (19.2%)	10 (8.0%)		
2	149	115 (77.2%)	28 (18.8%)	6 (4.0%)		
≥3	154	114 (74.0%)	33 (21.4%)	7 (4.5%)		
Frequency of visits by children or family/friends					H = 9.14	**0.0103**
Never	9	7 (77.8%)	1 (11.1%)	1 (11.1%)		
Rarely	35	22 (62.9%)	12 (34.3%)	1 (2.9%)		
Occasionally	55	38 (69.1%)	15 (27.3%)	2 (3.6%)		
Sometimes	128	91 (71.1%)	27 (21.1%)	10 (7.8%)		
Frequently	216	176 (81.5%)	31 (14.4%)	9 (4.2%)		
Type of older adult care institution					*χ*^2^ = 5.43	0.2289
Publicly built, privately operated	163	119 (73.0%)	32 (19.6%)	12 (7.4%)		
Publicly built, publicly operated	92	66 (71.7%)	23 (25.0%)	3 (3.3%)		
Privately built, privately operated	188	149 (79.3%)	31 (16.5%)	8 (4.3%)		
Gender					*χ*^2^ = 5.24	0.0795
Male	154	121 (78.6%)	22 (14.3%)	11 (7.1%)		
Female	289	213 (73.7%)	64 (22.1%)	12 (4.2%)		
Religious belief					*χ*^2^ = 5.65	0.0570
No	395	304 (77.0%)	73 (18.5%)	18 (4.6%)		
Yes	48	30 (62.5%)	13 (27.1%)	5 (10.4%)		
Marital status					*χ*^2^ = 5.14	0.4973
Married, spouse alive	130	99 (76.2%)	24 (18.5%)	7 (5.4%)		
Divorced	4	2 (50.0%)	1 (25.0%)	1 (25.0%)		
Widowed	298	223 (74.8%)	60 (20.1%)	15 (5.0%)		
Never married	11	10 (90.9%)	1 (9.1%)	0 (0.0%)		
Pre-retirement occupation					*χ*^2^ = 42.48	**0.0010**
Worker	183	145 (79.2%)	29 (15.8%)	9 (4.9%)		
Service industry	17	10 (58.8%)	2 (11.8%)	5 (29.4%)		
Teacher	27	20 (74.1%)	4 (14.8%)	3 (11.1%)		
Farmer	85	66 (77.6%)	18 (21.2%)	1 (1.2%)		
Other	24	20 (83.3%)	3 (12.5%)	1 (4.2%)		
Enterprise/Institution staff	85	57 (67.1%)	26 (30.6%)	2 (2.4%)		
Unemployed	8	5 (62.5%)	1 (12.5%)	2 (25.0%)		
Healthcare professional	14	11 (78.6%)	3 (21.4%)	0 (0.0%)		
Primary source of income					χ^2^ = 4.67	0.3068
Own income	288	211 (73.3%)	63 (21.9%)	14 (4.9%)		
Both	128	104 (81.2%)	17 (13.3%)	7 (5.5%)		
Children or relatives’ support	27	19 (70.4%)	6 (22.2%)	2 (7.4%)		
Reason for admission					*χ*^2^ = 2.77	0.6087
Realistic compromise	264	198 (75.0%)	51 (19.3%)	15 (5.7%)		
Passive placement	92	66 (71.7%)	20 (21.7%)	6 (6.5%)		
Active choice	87	70 (80.5%)	15 (17.2%)	2 (2.3%)		
Gender of living children					*χ*^2^ = 7.40	0.2799
Male	103	79 (76.7%)	18 (17.5%)	6 (5.8%)		
Both	224	170 (75.9%)	46 (20.5%)	8 (3.6%)		
Female	101	71 (70.3%)	21 (20.8%)	9 (8.9%)		
No children	15	14 (93.3%)	1 (6.7%)	0 (0.0%)		
Type of basic medical insurance					*χ*^2^ = 8.02	0.0915
Employee medical insurance/labor insurance	314	242 (77.1%)	61 (19.4%)	11 (3.5%)		
Resident medical insurance	123	88 (71.5%)	23 (18.7%)	12 (9.8%)		
Non-local medical insurance	6	4 (66.7%)	2 (33.3%)	0 (0.0%)		

C1 = Low-demand expression group; C2 = Multidimensional high-demand group; C3 = Psychological-prominent demand group. H indicates the Kruskal-Wallis H test statistic; *χ*^2^ indicates the Chi-square test statistic. Continuous variables are presented as median (P_25_, P_75_) due to non-normal distribution. Bold *p*-values indicate *p* < 0.10, which was considered statistically significant for screening candidate variables.

### 3.4 Multivariate analysis of factors influencing care demands profiles of disabled older adults

Using the care demands profiles of disabled older adults as the dependent variable (with C1 Low-demand expression group as the reference), variables with statistically significant differences in the univariate analysis were included as independent variables in multinomial logistic regression analysis. Results showed that age, pre-retirement occupation, frequency of visits by children or family/friends, number of chronic diseases, number of medications taken, religious belief, gender, and type of basic medical insurance all entered the regression equation. Variable assignments are shown in Table 4. Regression results are presented in Table 5.

**Table 4:** Variable assignment.

Variable name*	Assignment and meaning
Gender	0 = “Male”, 1 = “Female”
Religious belief	0 = “No”, 1 = “Yes”
Grade of older adult care institution	1 = “Level 1”, 2 = “Level 2”, 3 = “Level 3”, 4 = “Level 4”, 5 = “Level 5”
Care level of older adult	1 = “Mild”, 2 = “Moderate I”, 3 = “Moderate II”, 4 = “Severe I”, 5 = “Severe II”
Educational level	1 = “Illiterate”, 2 = “Primary school”, 3 = “Middle school”, 4 = “High school/technical secondary”, 5 = “Junior college”, 6 = “Bachelor’s degree”, 7 = “Master’s degree or above”
Number of chronic diseases	1 = “None”, 2 = “1 type”, 3 = “2 types”, 4 = “≥3 types”
Number of medications taken	1 = “0 types,” 2 = “1–3 types,” 3 = “3–5 types,” 4 = “≥5 types”
Length of stay in institution	1 = “<1 year,” 2 = “1–2 years,” 3 = “≥2 years”
Number of living children	1 = “0,” 2 = “1,” 3 = “2,” 4 = “≥3”
Frequency of visits by children or family/friends	1 = “Never,” 2 = “Rarely,” 3 = “Occasionally,” 4 = “Sometimes,” 5 = “Frequently”

*Before incorporating continuous variables, the linear relationships with the Logit function were tested using the Box-Tidwell method. All variables satisfied the linearity assumption and were directly included in the model. For unordered multicategorical variables, select a reference category and set dummy variables.

**Table 5:** Multivariate analysis of factors influencing care demands profiles of disabled older adults.

Variable	C2 vs C1		C3 vs C1	
	OR (95% CI)	*p* value	OR (95% CI)	*p* value
Age (per 1-year increase)	1.06 (1.01–1.11)	**0.013**	0.95 (0.89–1.01)	0.117
Pre-retirement occupation (reference: worker)				
Enterprise/institution staff	2.81 (1.43–5.51)	**0.003**	0.99 (0.18–5.42)	0.992
Service industry	0.81 (0.10–6.89)	0.846	0.59 (0.09–3.74)	0.577
Teacher	0.86 (0.26–2.90)	0.81	4.29 (0.92–20.00)	0.064
Farmer	0.98 (0.21–4.69)	0.983	0.02 (0.00–0.27)	**0.003**
Other	0.86 (0.22–3.27)	0.82	0.26 (0.03–2.79)	0.268
Unemployed	1.33 (0.09–18.70)	0.835	0.31 (0.03–3.49)	0.345
Healthcare professional	1.30 (0.33–5.15)	0.709	—	—
Frequency of visits by children or family/friends (per 1-level increase)	0.67 (0.53–0.85)	**0.001**	1.18 (0.74–1.90)	0.49
Number of chronic diseases (per 1-type increase)	0.99 (0.71–1.38)	0.959	0.62 (0.34–1.11)	0.107
Number of medications taken (per 1-level increase)	1.23 (0.84–1.79)	0.292	0.93 (0.42–2.05)	0.854
Religious belief (reference: No)				
Yes	1.72 (0.79–3.73)	0.173	3.79 (1.09–13.10)	**0.036**
Gender (reference: male)				
Female	2.00 (1.09–3.66)	**0.026**	0.42 (0.15–1.19)	0.102
Type of basic medical insurance (reference: employee medical insurance)				
Resident medical insurance	1.39 (0.30–6.40)	0.67	22.90 (4.34–120.00)	**<0.001**
Non-local medical insurance	1.92 (0.28–13.10)	0.504	—	—

C1 = Low-demand expression group (reference); C2 = Multidimensional high-demand group; C3 = Psychological-prominent demand group. OR = odds ratio; CI = confidence interval. The multivariate multinomial logistic regression model was adjusted for age, gender, pre-retirement occupation, frequency of children’s visits, number of chronic diseases, number of medications, religious belief, and type of basic medical insurance. Bold *p*-values indicate *p* < 0.05, which was considered statistically significant for screening candidate variables.

### 3.5 Network analysis of care demands of disabled older adults

#### 3.5.1 Overall network construction and Core demands

The overall network had 106 non-zero edges, with a network density of 41.9%. The top 5 demands items with the highest predictability (*R^2^*) were: RC16 (*R^2^* = 0.780), RC18 (*R^2^* = 0.776), LC4 (*R^2^* = 0.741), RC17 (*R^2^* = 0.735), and PC22 (*R^2^* = 0.664). The network structure is shown in Figure 2a. The correlation stability coefficient (CS-coefficient) indicated that the 95% confidence intervals for most edges were narrow and did not contain zero, and the original estimates were highly consistent with the bootstrap means, suggesting that the network structure was minimally affected by sampling variability. Stability testing of network centrality indices showed that both strength centrality (*CS* = 0.517) and expected influence (*CS* = 0.673) were >0.50, indicating that the network structure was stable against sample fluctuations and that the ranking of demands importance was highly reliable, as shown in Figure 2b.

**Figure 2:**
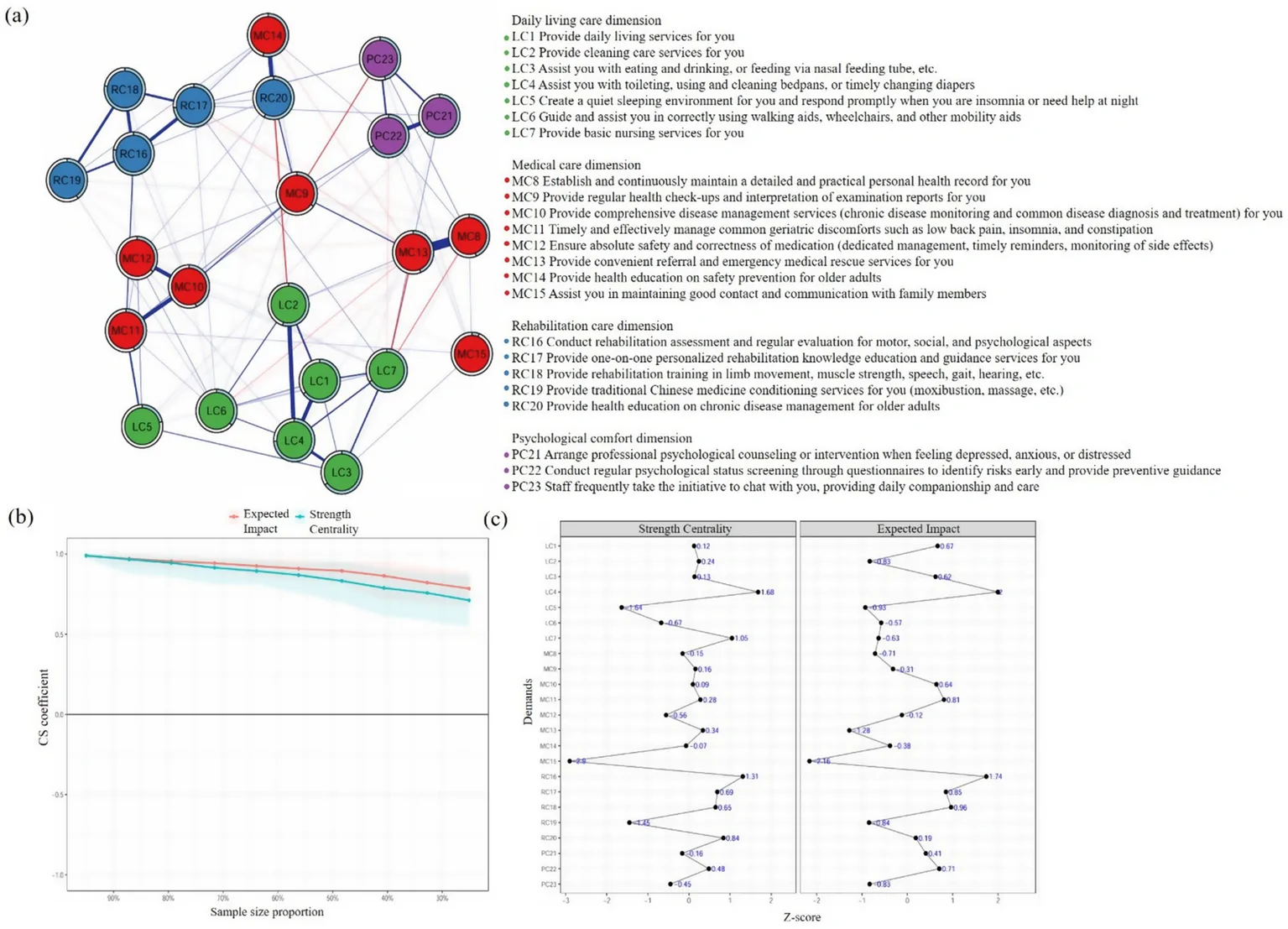
Network Analysis Diagram of Care demands for Fully Disabled Older adult **(a)** Care demands network diagram; **(b)** Stability testing of centrality indices; **(c)** Centrality indices of the care demands network.

Since the network contained both positive and negative edge weights, expected influence was used to measure the importance of demands in the network. The top 5 demands were: MC15, LC4, RC16, MC13, RC18, as shown in Figure 2c. The top 5 demands with the highest bridge expected influence were: RC20 (*BEI* = 0.663), MC14 (*BEI* = 0.661), MC11 (*BEI* = 0.525), LC5 (*BEI* = 0.362), and MC12 (*BEI* = 0.306).

#### 3.5.2 Subgroup network analysis

To further reveal demands heterogeneity, subgroup network analyses were conducted separately for the C1 Low-demand expression group (*n* = 334) and the C2 Multidimensional high-demand group (*n* = 86) (C3 sample size was only 23 and was not included), as shown in Figure 3 and Figure 4. Across both profiles, the nodes with the highest expected impact are assisting family communication (PC23) and excretion care services (LC4). In C1, the strongest positive edges were between safety prevention services (MC14) and chronic disease health education (RC20) (0.525), rehabilitation assessment services (RC16) and rehabilitation training services (RC18) (0.423), and emergency rescue services (MC13) and health record establishment services (MC8) (0.415). In C2, the strongest positive edges were between rehabilitation assessment services (RC16) and rehabilitation training services (RC18) (0.549), emergency rescue services (MC13) and health record establishment services (MC8) (0.513), and disease diagnosis and treatment services (MC10) and medical treatment services (MC11) (0.410). Across both profiles, excretion care services (LC4) and dietary care services (LC3) were positively correlated, rehabilitation assessment services (RC16) and rehabilitation training services (RC18) were positively correlated, emergency rescue services (MC13) and health record establishment services (MC8) were positively correlated, and safety prevention services (MC14) and cleaning care services (LC2) were negatively correlated.

**Figure 3:**
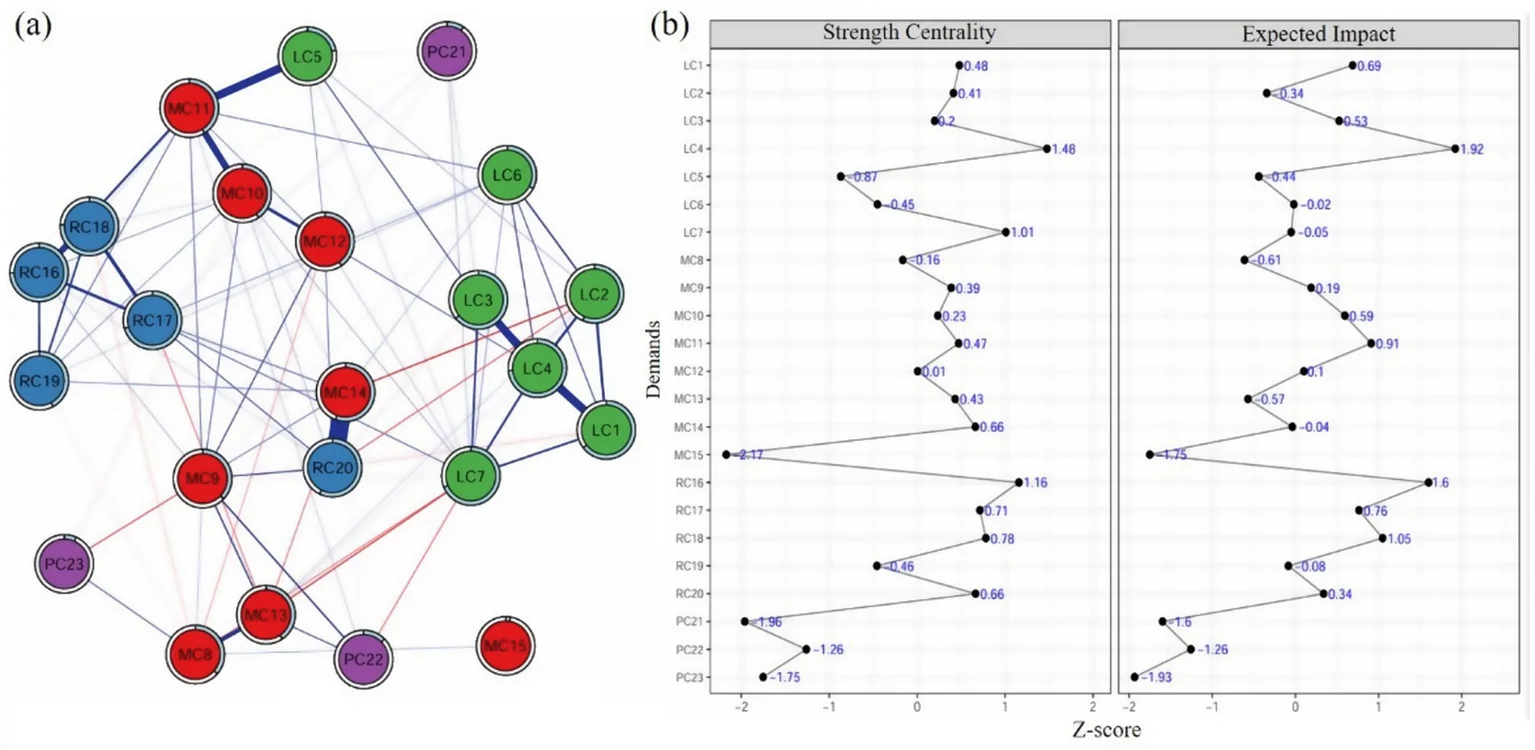
Network Analysis Diagram of Care demands for Disabled Older adult with Low Care Demands **(a)** Care demands network diagram of the Low-demand expression group; **(b)** Centrality indices of the care demands network of the Low-demand expression group.

**Figure 4:**
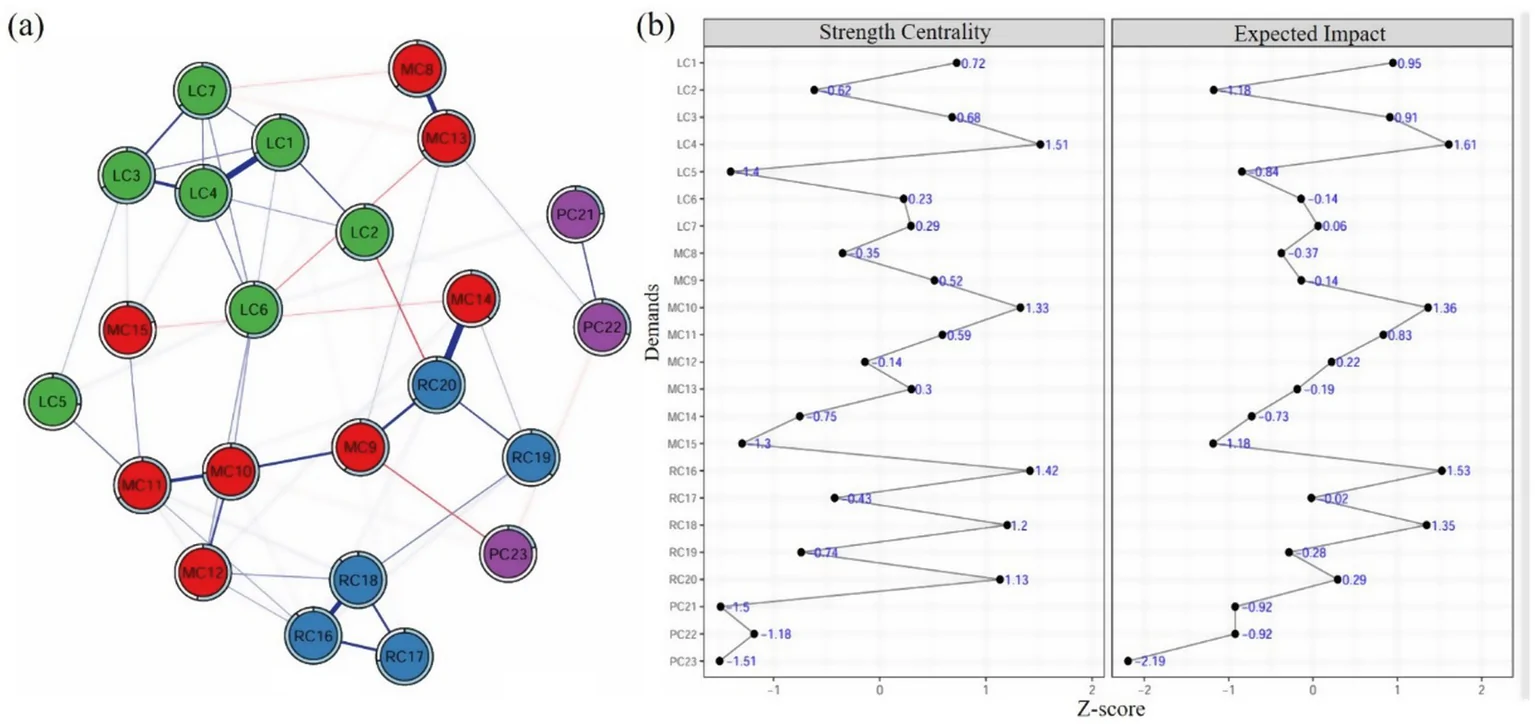
Network Analysis Diagram of Care demands for Disabled Older adult in Multidimensional high-demand group **(a)** Care demands network diagram of the Multidimensional high-demand group; **(b)** Centrality indices of the care demands network of the Multidimensional high-demand group.

## 4 Discussion

### 4.1 Heterogeneity of care demands among disabled older adults in integrated medical–nursing care institutions

In this study, the medical care dimension scored the highest (median = 3.12, IQR: 2.62–3.38) and the psychological consolation dimension the lowest (median = 1.33, IQR: 1.00–2.00) among disabled older adults in integrated medical–nursing care institutions, which is consistent with the findings of (20) in similar settings. This reflects that current institutions place relatively greater emphasis on the provision of medical care services, whereas psychological consolation may have considerable room for improvement. However, dimensional mean scores only reflect overall tendencies at the group level and cannot capture inter-individual differences in demands patterns. Through latent profile analysis from a person-centred perspective, this study identified three demand subtypes: the “low care demands” group (76.4%), the “comprehensive care demands” group (18.5%), and the “high psychological care demands” group (5.1%), indicating that the care demands of disabled older adults are clearly typologically differentiated. ① The “low care demands” group (76.4%) had lower mean scores across all dimensions than the other two groups and accounted for the vast majority. This distribution may be explained by two mechanisms. First, under the institution’s provider-led care model, disabled older adults have long been in a passive recipient role; they become accustomed to being arranged for and having decisions made on their behalf, which weakens their initiative to express their own demands (35). Second, older adults may have only vague perceptions of what institutional services can offer—particularly regarding the scope and potential of non-basic services such as psychological consolation and rehabilitation training—making it difficult for them to form clear expectations, and thus they tend to choose lower response options in demands assessments. However, low demand expression does not equate to an absence of needs; long-term concealment of demands may lead to accumulation of health problems. ② The “comprehensive care demands” group (18.5%) had higher mean scores in the daily living care, medical care, and rehabilitation care dimensions than the other two groups, with psychological consolation demands in the middle, indicating that this group’s reliance on institutional services is multifaceted. For this group, a single, fragmented service delivery model may not effectively meet their care demands. ③ The prominent feature of the “high psychological care demands” group (5.1%) was that the mean score of the psychological consolation dimension was higher than that of the other two groups, while the demands levels in the other three dimensions were relatively low. The independent existence of this subtype suggests that psychological care demands are somewhat independent in some disabled older adults and do not necessarily arise solely with physical dysfunction. Based on the above findings, institutions may consider paying closer attention to the potential care demands of older adults in the low-demand group, conducting regular proactive screening using standardized assessment tools, and helping older adults gain a better understanding of various care services through service introductions and experiential activities, thereby gradually fostering their proactive demand-expressing behaviors. For the Multidimensional high-demand group, a potential direction for service improvement could be the exploration of multidisciplinary team-based case management pathways. For the Psychological-prominent demand group, preliminary evidence suggests that strengthening the allocation of psychological counseling or mental health service resources may be worth considering. In addition, the multivariate analysis of this study showed that the frequency of children’s visits was associated with profile membership, suggesting that family support plays a role for this group. Institutions may consider building an “institution-family” communication bridge, encouraging family members to increase visit frequency and maintain daily contact through video calls and other means, to partially compensate for the shortage of institutional psychological care resources. However, these suggestions are derived from cross-sectional observational data and should be interpreted as hypothesis-generating rather than evidence-based recommendations for practice. Future interventional studies are needed to evaluate the effectiveness of these strategies.

### 4.2 Factors influencing the latent profiles of care demands among disabled older adults

#### 4.2.1 Influence of predisposing factors among personal characteristics

The results showed that age, gender, and pre-retirement occupation were influencing factors for latent profile membership among disabled older adults (all *P*<0.05). Each additional year of age was associated with an approximately 6% increase in the odds of belonging to the comprehensive care demands group (OR = 1.06), suggesting that age-related physiological decline and aggravation of disability are possible factors driving comprehensive care demands. Regarding gender, women had a higher risk than men of being in the Multidimensional high-demand group (*OR* = 2.00, *95%CI*: 1.09–3.66, *p* = 0.026), possibly because women are more inclined to actively voice physical discomfort and psychological concerns. Those who had been enterprise or institution staff before retirement showed a higher risk than workers (*OR* = 2.81, *95%CI*: 1.43–5.51, *p* = 0.003); the mechanism may relate to higher health service expectations, more systematic health literacy, and relatively greater financial reserves formed during their employment, leading to more refined demands for service content and quality after entering institutions. Educational level, marital status, number of living children, and gender of living children were not associated with profile membership in this study, which may be due to the fact that the majority of the sample were married or had children, with relatively balanced distribution across groups. These findings suggest that institutions may consider age, gender, and occupational background as reference factors in care planning to facilitate early identification of those with complex care needs.

#### 4.2.2 Influence of enabling factors among personal characteristics

The results showed that frequency of visits by children or family/friends was an independent influencing factor for latent profile membership (*P*<0.05). For each one-level increase in visiting frequency, the risk of belonging to the Multidimensional high-demand group decreased by approximately 33% (*OR* = 0.67, *95%CI*: 0.53–0.85, *p* = 0.001). Although the univariate analysis of Zhao et al. (36) suggested that visiting frequency was associated with care demands scores, their multivariate regression did not confirm it as an independent factor; Zhang et al. (37).did not find a significant effect of visiting frequency on long-term care satisfaction, possibly due to differences in outcome variables (total demand score/satisfaction) in those studies, whereas this study, based on latent profile analysis, further clarified its independent role in demand typing. Frequent family visits not only provide emotional support and information transmission but may also assist institutional caregivers in promptly understanding changes in the older adults’ needs, serving as a supplement to institutional care. Regarding medical insurance type, the association between resident medical insurance and the Psychological-prominent demand group was estimated with limited precision (OR = 22.90, 95% CI: 4.34–120.00). The high psychological care demands subgroup contained only 23 cases, of whom 12 (52.2%) held resident medical insurance, whereas the corresponding proportion in the Low-demand expression group (reference) was 22.5% (see Table 3). Given this substantial imbalance and the limited subgroup sample size, the estimate is statistically unstable and lacks sufficient precision for causal inference. We therefore refrain from interpreting this association substantively and report it descriptively only. Personal monthly income, source of income, and length of stay in the institution were not associated with profile membership in this study, possibly due to the relatively homogeneous income levels and income source structures in the sample. It is suggested that institutions incorporate family visiting frequency into needs assessment considerations; for those with low visiting frequency, institutions may compensate for insufficient social support by increasing communication frequency and providing psychological counselling.

#### 4.2.3 Influence of need factors and contextual factors among personal characteristics

At the level of need factors, number of chronic diseases, number of medications taken, care level, and reason for admission did not enter the multivariate regression equation (all *p* > 0.05 in univariate analysis; Table **3**). Among these, care level showed no significant association with profile membership (*p* = 0.6610). In this study, we observed that some older adults with severe disability were classified into the “low care demands” group, while some with mild disability belonged to the “comprehensive care demands” or “high psychological care demands” groups. This pattern suggests that the differentiation of care demand profiles is not simply determined by the degree of physical disability; rather, demand expression is also moderated by factors such as individuals’ willingness to voice their needs, their awareness of available institutional services, their psychological adaptation status, and their prior experiences with family care. In addition, the severe imbalance in subgroup sample sizes (C3: *n* = 23) may have further limited the statistical sensitivity of tests for need-related factors. In summary, it is not that need factors are unrelated to care demand profiles; rather, their association in the institutional care context is likely mediated or moderated by multiple psychosocial factors, and the limited subgroup sample sizes may have attenuated the statistical power to detect between-group differences. At the contextual level, institution type (publicly built and privately operated, publicly built and publicly operated, privately built and privately operated) and institution grade also showed no significant association with profile membership (all *p* > 0.05), with similar distributions across the three groups. This does not exclude the possibility that different patterns might emerge in other regions or more diverse institutional samples (18), however, within the present sample, these variables did not exhibit detectable discriminating power.

### 4.3 Core structure of the care demands network and implications for service provision

Network analysis revealed the inter-item association structure of care demands among disabled older adults from the perspective of conditional dependencies. Beyond reflecting the independent intensity of each demand, centrality indices identify nodes that may play structurally dominant roles in the system.

In the overall network, “excretion care” (LC4) and “rehabilitation assessment” (RC16) ranked second and third in expected influence centrality, suggesting that they may serve as hubs to some extent. Specifically, “excretion care” was associated with basic daily living care items such as dietary care and daily living assistance; “rehabilitation assessment” is the entry point for initiating rehabilitation training, guidance, and outcome evaluation. This finding implies that institutions may prioritise ensuring the accessibility of services with high centrality, thereby leveraging limited resources to drive coordinated responses across multiple demand dimensions. In the overall network, “chronic disease management health education” (RC20) and “safety prevention health education” (MC14) were identified as bridge demands—that is, strengthening the provision of these two services may simultaneously activate responses across several dimensions. However, (32) found that these two services had the largest supply–demand gaps in current integrated medical–nursing care institutions; (38) also reported significant discrepancies between high demand scores and low supply scores for “regular chronic disease lectures” and “using multiple media to disseminate health knowledge.” (39) demonstrated that individualised health education based on a knowledge-base model (delivered once every 3 months) could effectively improve health literacy among middle-aged and older adults with chronic conditions. Integrated medical–nursing care institutions could adopt this intervention approach by upgrading routine health education to periodic, personalised health information delivery, thereby precisely addressing the core demands of disabled older adults in chronic disease management and safety protection and bridging the structural supply–demand gap. However, as the present study is cross-sectional in design, these implications remain preliminary and require confirmation through interventional or longitudinal studies.

This study also found differences in network structure between the two subgroups: in the “low care demands” group, the strongest edge was the cross-dimensional connection between “safety prevention services” (MC14) and “chronic disease health education” (RC20), suggesting that demands cognition in this group may be relatively diffuse; in the “comprehensive care demands” group, the strongest edges were concentrated within each dimension, such as strong connections between “rehabilitation assessment” (RC16) and “rehabilitation training” (RC18), and between “emergency rescue services” (MC13) and “health record establishment” (MC8), indicating a more modular structure of demands. This structural difference has practical implications for service delivery strategies: for the low-demand group with diffuse cognition, a comprehensive assessment system could be prioritised to systematically identify potential multidimensional needs; for the comprehensive-demand group with modularised structures, attention should be paid to deepening service depth and continuity within each core domain to enhance the precision of resource allocation. It should be noted that the “comprehensive care demands” group had a sample size of 86, and the network stability test yielded a CS coefficient of 0.279 for strength centrality and 0.442 for expected influence, with both values falling between 0.25 and 0.5 (34)., the results of this subgroup network analysis should be further validated in larger samples.

## 5 Limitation

This study has several limitations. First, convenience sampling was used and the sample was drawn solely from 16 institutions in Pudong New Area, Shanghai, limiting generalizability. Second, the cross-sectional design provides only a snapshot of care demands at a single time point and does not capture their dynamic evolution over time. Third, certain subgroups had limited sample sizes (e.g., Psychological-prominent demand group, *n* = 23), resulting in limited statistical precision; these regression results are presented descriptively. Fourth, subgroup network analyses were constrained by sample size (Multidimensional high-demand group, *n* = 86), with CS coefficients falling within the acceptable range (0.25–0.5) but below the 0.5 threshold for good stability; these results should be interpreted with caution. Fifth, service supply data were not included, and demand expression may be constrained by service availability. Future research with larger and more diverse samples, incorporating longitudinal designs and supply–demand matching data, is warranted to further validate these findings.

## Data Availability

The original contributions presented in the study are included in the article/supplementary material, further inquiries can be directed to the corresponding author.
